# Optical coherence tomography angiography macular biomarkers of peripheral retinal ischemia in diabetic macular edema: secondary endpoints from the clinical study “FOVEA”

**DOI:** 10.1007/s00417-024-06372-6

**Published:** 2024-01-20

**Authors:** Rita Serra, Florence Coscas, Jean François Boulet, Diogo Cabral, Thi Ha Chau Tran, Antonio Pinna, Marco Lupidi, Gabriel Coscas, Fovea study group, Pierre-Loic Cornut, Joel Uzzan, Flore DeBats, Jean-Philippe Theron, Benjamin Wolff, Catherine Francais, Catherine Favard

**Affiliations:** 1 Department of Medicine, Surgery and Pharmacy, Ophthalmology Unit, University of Sassari, Sassari, Italy; 2 Istituto di Ricerca Genetica e Biomedica (IRGB), CNR, Cittadella Universitaria di Cagliari, (CA), 09042 Monserrato, Italy; 3 Centre Ophtalmologique de l’Odéon, 113 bd Saint Germain, Paris, France; 4 Paris VI University, 361 rue Clément Ader, Bâtiment C, 27000 Evreux, France; 5 Instituto de Oftalmologia Dr. Gama Pinto, Lisbon, Portugal; 6 Ophthalmology Department, Lille Catholic Hospitals, Lille Catholic University, INSERM, U1172, Lille, France; 7 Eye Clinic, Department of Experimental and Clinical Medicine, Polytechnic University of Marche, Ancona, Italy

**Keywords:** Diabetic macular edema, Fractal dimension, Lacunarity, Peripheral retinal ischemia

## Abstract

**Purpose:**

To investigate the relationship between the macular values of fractal dimension (FD) and lacunarity (LAC) on optical coherence tomography angiography (OCTA) images and the presence of peripheral retina non-perfusion areas (NPAs) on fluorescein angiography (FA) in patients with treatment-naïve diabetic macular edema (DME).

**Methods:**

Fifty patients with treatment-naïve DME underwent a full ophthalmic examination, including best-corrected visual acuity measurement, FA, spectral-domain optical coherence tomography, and OCTA. Specifically, FA was performed to detect the presence of retinal NPAs, whereas fractal OCTA analysis was used to determine macular FD and LAC values at the level of the superficial and deep capillary plexus (SCP and DCP). FA montage frames of the posterior pole and peripheral retina, as well as macular OCTA slabs of the SCP and DCP, were obtained.

**Results:**

Thirty (60%) eyes with FA evidence of peripheral retinal NPAs in at least one quadrant showed significantly lower FD and higher LAC in both SCP and DCP, when compared with eyes presenting a well-perfused peripheral retina. Furthermore, macular FD and LAC values were found to be significantly associated with the extent of retinal NPAs.

**Conclusions:**

Macular FD and LAC of both SCP and DCP seem to be strongly associated with the extent of peripheral retinal NPAs, thus suggesting that may be useful predictive biomarkers of peripheral ischemia in treatment-naïve DME eyes.


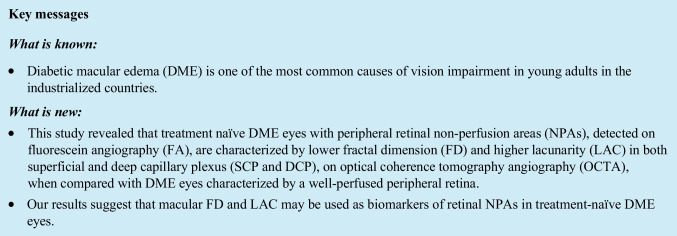


## Introduction

Diabetic retinopathy (DR), the most common complication of diabetes mellitus, is one the main causes of vision impairment in the adult working population and the elderly [1].

DR is a microangiopathy characterized in the early stages by microaneurysms and punctate hemorrhages, which can evolve into more severe forms complicated by diabetic macular edema (DME), retinal non-perfusion areas (NPAs), and neovascularization [2].

Traditionally, the early detection of DR features relies on fluorescein angiography (FA), an essential tool in grading DR severity and detecting NPAs and retinal neovascularization.

Although FA introduction dates to more than 6 decades ago, it remains the gold standard in the diagnosis and grading of DR [3]. Nevertheless, FA is an invasive procedure, expensive, time-consuming, and uncomfortable for the patient, with documented risks of possible allergic reactions.

Furthermore, mydriatic eye drops instillation is mandatory to perform FA and the quality of images obtained is strongly dependent on the photographer’s technical skills, patient’s cooperation, and ocular media opacities [3, 4].

In the last years, optical coherence tomography angiography (OCTA), an imaging technique providing high-resolution scans of the retinal vasculature layers in a few seconds, has become more and more popular in clinical settings. Unlike FA, OCTA is dye-free and allows the visualization of all the relevant vascular layers in the retina [5].

Emerging evidence indicates that the retinal vascular tree, showing a branching pattern with self-similarity properties, is a fractal structure and can, therefore, be evaluated by fractal analysis [6].

OCTA fractal analysis allows to estimate quantitative parameters providing insights into the architecture and vascular complexity of neovascular age-related macular degeneration (AMD) [5]. Recently, several studies have been performed to identify possible OCTA biomarkers of DR severity [6–9]; however, the results reported have been controversial.

The purpose of this study was to investigate fractal quantitative OCTA parameters, such as macular fractal dimension (FD) and lacunarity (LAC), in treatment-naïve DME eyes and to assess the relationship between FD and LAC and the presence of retinal NPAs, in order to identify predictors of peripheral ischemia.

## Methods

This was a multicenter retrospective observational study based on a pool of treatment-naïve eyes with non-proliferative diabetic retinopathy (NPDR) enrolled at seven high-volume referral centers, members of the FOVEA study group, between February 2017 and March 2018.

The inclusion criterion was the presence of treatment-naïve DME in NPDR patients with type 1 or 2 diabetes mellitus.

DME was defined as a macular thickening due to the accumulation of intra- and/or sub-retinal fluid on spectral-domain optical coherence tomography (SD-OCT), secondary to blood-retinal barrier (BRB) breakdown with dye leakage on FA [10].

The exclusion criteria were poor-quality images on traditional multimodal retinal imaging and/or OCTA. Furthermore, eyes with ophthalmologic diseases other than DR (e.g., AMD, uveitis, glaucoma, epiretinal membrane, retinal vein or artery occlusion), previous surgery (including cataract surgery within 6 months before recruitment), and retinal laser photocoagulation, as well as history of intravitreal injections with anti-vascular endothelial growth factor (VEGF) agents or steroids, were excluded.

The present study was conducted in compliance with tenets of the Declaration of Helsinki for research involving human subjects and approved by the Paris Review Committee (Protocol n. 2017-A00383-50). Written informed consent was obtained by each participant.

### Ophthalmologic examination

All patients underwent an extensive ophthalmic examination and retinal imaging, including measurement of best-corrected visual acuity (BCVA) using Early Treatment Diabetic Retinopathy Study (ETDRS) charts, slit-lamp biomicroscopy with dilated indirect fundoscopy, SD-OCT (SD-OCT; Spectralis; Heidelberg Engineering, Heidelberg, Germany), and FA (Spectralis HRA + OCT; Heidelberg Engineering, Heidelberg, Germany). Retinal images were obtained after pupil dilation with 1% tropicamide eye drops to improve image quality.

Distribution of DME (intraretinal vs. sub-retinal fluid) as well as the presence of macular exudates was evaluated on SD-OCT by a macular volume scan (49 high-resolution B-scans within a 20° × 20° area) centered on the fovea.

The retinal map analysis protocol of the SD-OCT was used to assess retinal thickness in the nine ETDRS sectors. The inner and outer rings were segmented into four quadrants, with radii of 1.5 and 3 mm, respectively, whereas foveal thickness was defined as the average thickness in the central 1000-μm diameter of the ETDRS layout [11].

FA was performed using a 55° lens for the posterior pole and for the periphery (the patient was asked to look superiorly, inferiorly, nasally, and temporally to assess the extreme retinal periphery). Two masked retinal specialists (RS and FC) analyzed FA images to detect retinal NPAs in the four peripheral quadrants (superior, inferior, nasal, and temporal). NPA was defined as a retinal area characterized by the absence of visible arterioles and/or capillaries with hypo-fluorescence relative to the overall background on a mid-phase FA frame [12]. Rare disagreements over readings were resolved by open adjudication between readers.

Macular OCTA scans were obtained, centering the volume scan on the foveal center.

The in-built software (SP-X2001 Update 3, based on Heyex Software Version 1.9.215.0H, Heidelberg Engineering, Heidelberg, Germany) is equipped with a projection artifact removal (PAR) tool, which automatically removes any shadowgraphic artifact from the selected C-scan. Particularly, PAR is defined as the obscuration of vessels in deeper layers due to the removal of projected superficial vessels by the device software. The segmentation strategies aimed to ensure a proper positioning of the slab edges have been described previously [13].

For correct visualization of the capillary plexuses and removal of artifacts potentially confounding image interpretation, automatic segmentation was checked and manually adjusted, if needed, by an expert retinal specialist (FC). Manual adjustment of the thickness between two segmentations is of critical importance to improve the visualization of the vascular plexus. OCTA images affected by artifacts were not considered for further analysis [14].

Then, 3 × 3 OCTA scans of the SCP and DCP were exported into a previously validated custom graphical interface, created by means of MATLAB (v.r2018a) coding language, to perform fractal analysis of the macular slabs [5, 15].

OCTA images were binarized by using the Otsu method, and a median filter of a radius of two pixels was applied to remove speckle noise, allowing an optimal threshold value of the input image, as previously reported [15].

Then, binarized OCTA images were skeletonized to calculate FD and LAC, which are, respectively, global indices of morphological complexity and structural nonuniformity. Noteworthy, FD is a statistical descriptor of space-filling patterns that allows evaluation of both the architecture and the degree of pattern complexity of the vascular network. Box counting method at multiple origins was used for FD measurement. Specifically, images were divided into square boxes of equal size, and the number of boxes containing a vessel segment was computed for FD measurement. Therefore, according to vessel distribution, the FD value ranged from 0 to 2.

On the other hand, LAC, by definition the counterpart to FD, describes the texture of a fractal and measures the nonuniformity of a vascular network, thus making it possible to distinguish sets with the same FD, but different textures. LAC was estimated by means of mathematical formulae assessing the pixel distribution in binary skeleton images to evaluate image heterogeneity [5, 14, 15].

### Statistical analysis

The results of descriptive analysis are reported as numbers and percentages for categorical variables and as means ± standard deviation (SD) for quantitative variables. After testing the data distribution for normality, *t*-test was used, as appropriate. The relationship between macular FD and LAC values on OCTA and NPAs on FA was evaluated using Pearson’s correlation test. A *p* value of <0.05 was considered statistically significant. The study data were analyzed using the Statistical Package for Social Sciences version 20.0 for Mac (IBM, Chicago, IL, USA).

## Results

Fifty treatment-naïve DME eyes of 50 NPDR patients (25 men, 25 women, mean age 66.44±10.66 years) included in the clinical study “FOVEA” were evaluated. Twenty-four (48%) of them (13 men, 11 women; mean age 64.83±12.1 years) had type 1 diabetes, whereas the remaining 26 (52%; 12 men, 14 women; mean age 67.92±9.12 years) had type 2 diabetes. No significant differences between the two groups were found in terms of age and gender.

Overall, the mean BCVA was 63.04±13.13 ETDRS letters, whereas the mean central macular thickness (CMT) was 430.02±109.36 μm.

SD-OCT revealed the presence of sub-retinal fluid in 14/50 (28%) eyes, whereas macular exudates were found in 22/50 (44%) eyes.

FA disclosed that 20/50 (40%) eyes had a well-perfused retina, whereas the remaining 30/50 (60%) showed NPAs. Specifically, when evaluating the four peripheral quadrants, 11/30 (36.67%) eyes presented NPAs in all peripheral quadrants, 9/30 (30%) in three peripheral quadrants, 4/30 (13.33%) in two peripheral quadrants, and the remaining 6/30 (20%) in only one peripheral quadrant.

Overall, mean FD and LAC were 1.6±0.53 and 0.35±0.08, respectively, at the level of the SCP and 1.63±0.45 and 0.36±0.07, respectively, at the level of the DCP. No significant difference was found between SCP and DCP in terms of FD and LAC.

All demographic, clinical, and OCTA data are summarized in Table 1.

**Table 1:** Demographic, clinical, and OCTA features in patients with diabetic macular edema presenting retinal non-perfusion areas (NPAs) or a well-perfused retina

	Eyes with retinal NPAs	Eyes with a well-perfused retina	*p value*
Eyes, *n* (%)	30 (60%)	20 (40%)	>0.05
Gender			
Male, *n* (%)	16 (53%)	11 (55%)	>0.05
Female, *n* (%)	14 (47%)	9 (45%)	>0.05
Age, mean ± SD (years)	63.8 ± 10.51	70.40 ± 9.84	>0.05
BCVA, mean ± SD (ETDRS letters)	61.83 ± 12.23	64.70 ± 12.64	>0.05
CMT, mean ± SD (μm)	442.13 ± 115.75	420.28 ± 106.02	>0.05
*OCTA fractal parameters*			
SCP			
FD, mean ± SD	1.21 ± 0.58	1.92 ± 0.03	0.0003
LAC, mean ± SD	0.39 ± 0.06	0.31 ± 0.08	0.006
DCP			
FD, mean ± SD	1.25 ± 0.40	1.94 ± 0.03	<0.0001
LAC, mean ± SD	0.38 ± 0.05	0.31 ± 0.06	0.0009

*BCVA* best-corrected visual acuity, *CMT* central macular thickness, *SCP* superficial capillary plexus, *DCP* deep capillary plexus, *FD* fractal dimension, *LAC* lacunarity

Eyes with FA evidence of peripheral retinal NPAs in at least one quadrant showed significantly lower FD and higher LAC in both the SCP and DCP, when compared with eyes presenting a well-perfused peripheral retina.

Macular FD values of both SCP and DCP were significantly related to NPAs (*r*=−0.83, *p*<0.0001 and *r*=−0.91, *p*<0.0001; respectively). Similarly, macular LAC values of both SCP and DCP were significantly related to NPAs (*r*=0.35; *p*=0.03 and *r*= 0.43; *p*= 0.007).

## Discussion

In the last few years, numerous authors have investigated the application of fractal analysis to DR and other retinal vascular disorders. Specifically, Bhardwaj et al. [9] have reported that FD values of both DCP and SCP are significantly lower in DR eyes than in healthy controls.

Similarly, in a retrospective observational study including 84 DR eyes and 14 healthy controls, Kim et al. [7] demonstrated that subjects with severe DR have significantly lower FD values at the level of the SCP, when compared to healthy eyes. Furthermore, these authors also found a negative correlation between DR severity and FD, a result suggesting that DR worsening may be associated with a decreased branching complexity [7].

Other studies have demonstrated that FD is positively correlated with increasing odds of DR; in particular, for each 0.01 increase of FD, there is a nearly 40% increase in the odds of DR, a finding supporting the idea that fractal analysis may be a new method to objectively assess DR-related vascular damage [6].

Likewise, Sun et al. [8], comparing eyes with and without DME, have shown that fractal parameters are correlated with DME. Indeed, in this prospective study assessing 205 eyes of 129 patients with a 2-year follow-up, SCP vascular density was found to be a biomarker predictive of DME occurrence [8].

Furthermore, decreased FD values at the level of both SCP and DCP have also been reported in other ocular vascular diseases, including retinal arterial and venous occlusions, non-arteritic anterior ischemic optic neuropathy, and macular telangiectasia type 2 [16–19].

In our study, we performed the fractal analysis of SCP and DCP OCTA slabs in treatment-naïve DME eyes and analyzed the correlation between FD and LAC and the presence of peripheral retinal NPAs on FA. Mean FD and LAC values were 1.60±0.53 and 0.35±0.08, respectively, at the level of SCP and 1.63±0.45 and 0.36±0.07, respectively, at the level of DCP.

Our FD macular results are consistent with those previously reported by Sun et al. [8] who found mean FD values of 1.68±0.05 (SCP) and 1.67±0.05 (DCP), with no significant difference between the two OCTA plexuses.

Conversely, in an OCTA study on DR investigating the macular changes in the superficial, intermediate, and deep plexus, Onishi et al. [20] observed a relative preservation of capillary flow in the SCP, compared with a steep decline in the intermediate and deep plexuses. By contrast, Falvarjani et al. [21], investigating the distribution of NPA in SCP and DCP plexus in a pool of 27 DR eyes, reported that NPA was higher in SCP than in DCP. Although these authors used several strategies to reduce the risk of artifacts, it is possible that NPA measurements may have been affected by projections artifacts, thus resulting in higher NPA in the SCP rather than in the DCP [21].

Indeed, histologic evidence supports the theory that the DCP is the most vulnerable to DR [20, 22]. Therefore, it is likely that microvascular DR changes may initially involve the DCP, with blood-retinal barrier (BRB) breakdown, accumulation of intraretinal fluid, and DME development, and only later affect the SCP.

Thus, it is not surprising that in our study, including patients with treatment-naïve DME, we failed to find any significant difference between the two capillary plexuses.

We found that eyes with FA evidence of peripheral retinal NPAs in at least one quadrant showed significantly lower FD and higher LAC in both the SCP and DCP, when compared with eyes presenting a well-perfused peripheral retina. Furthermore, FD and LAC values of both SCP and DCP were significantly related to the extent of retinal NPAs. Specifically, there was an inverse correlation between FD and LAC values which tended to decrease; that is, decreased FD values corresponded to LAC increase, and vice versa. All these data suggest that the perfusion status of the peripheral retina is strongly related to macular changes, objectively detectable by fractal analysis.

Theoretically, the extent of peripheral NPA might contribute to the degree of DR-related inflammation and retinal vascular alterations. Indeed, diabetic microangiopathy, including arterial constriction, microcapillary occlusion, and, therefore, reduced FD values, may promote NPA occurrence and trigger the inflammatory cascade with the release of several cytokines and VEGF, which synergistically promotes BRB breakdown, intraretinal fluid accumulation, DME, and, finally, increased LAC values [23].

Higher LAC values correspond to a higher degree of “gappines” and greater size distribution of the lacunae. Actually, DME appearance on OCTA is characterized by a bigger black cystoid area with no flow signal. Accordingly, the most severe forms of DME have bigger lacunae corresponding to higher LAC values.

Our results suggest that macular FD and LAC values may be potential biomarkers of severity of both DME and peripheral retinal ischemia in eyes with DR.

In a former survey assessing FD values in DR, Fan et al. [24] found that FD of the entire retina was strongly related to the extent of peripheral retinal ischemia. Similarly, Kaderli et al. [25], assessing 59 eyes with NPDR and 36 eyes with proliferative diabetic retinopathy (PDR), found that the foveal avascular zone was positively correlated with NPA in both groups. Furthermore, a negative correlation was found between parafoveal vascular density in SCP and DCP and NPA in NPDR eyes [25]. In PDR eyes, this negative correlation was found only for the DCP [25].

Consistently, Li et al. [26] have reported that the foveal flow area in the choroid capillary plexus was significantly lower in DR patients than in controls without DR. These authors suggest that in diabetic eyes, the microvascular ischemia originates in the choroid layer and extends inward into DCP and SCP and conclude that OCTA can serve as a reliable method for early detection and monitoring progression of DR [26]

In another study, Sekzaly et al. [12] demonstrated that macular OCTA parameters were significantly associated with the perfusion status of the peripheral retina in retinal vein occlusions. Specifically, macular vascular density was significantly related to the extent of peripheral retina ischemia, and a cut-off value distinguishing eyes with well-perfused peripheral retina from those showing peripheral NPA was also identified [12]. Similarly, other authors have highlighted the importance of OCTA quantitative parameters in differentiating central retinal vein occlusion from branch retinal vein occlusion [16].

Our study has some important limitations, including the relatively small sample size. Another important limitation is that FA image montage of standard frames was not standardized, which may have resulted in small differences between patients. Furthermore, although we manually adjusted the automatic segmentation of both plexuses, we cannot exclude that DME presence may have somehow affected the computation of the fractal macular parameters. Finally, we acknowledge that less important factors, such as image brightness and contrast, may have influenced FD computation [27]. Nevertheless, we are unaware of any previous survey applying fractal analysis to treatment naïve DME eyes and assessing macular FD and LAC values of both SCP and DCP.

In conclusion, our results indicate that macular FD and LAC are strongly related to the presence of peripheral NPAs, thus suggesting that they may be potential biomarkers of peripheral retinal ischemia. Further larger studies are warranted to confirm our preliminary findings, highlighting the potential role of macular fractal parameters in the prediction of peripheral NPAs, when FA is not feasible.
